# Activation Profile Analysis of CruCA4, an α-Carbonic Anhydrase Involved in Skeleton Formation of the Mediterranean Red Coral, *Corallium rubrum*

**DOI:** 10.3390/molecules23010066

**Published:** 2017-12-28

**Authors:** Sonia Del Prete, Daniela Vullo, Didier Zoccola, Sylvie Tambutté, Claudiu T. Supuran, Clemente Capasso

**Affiliations:** 1 Istituto di Bioscienze e Biorisorse, CNR, Via Pietro Castellino 111, 80131 Napoli, Italy; sonia.delprete@ibbr.cnr.it; 2 Dipartimento Di Chimica, Laboratorio di Chimica Bioinorganica, Università degli Studi di Firenze, Polo Scientifico, Via della Lastruccia 3, 50019 Sesto Fiorentino (Florence), Italy; daniela.vullo@unifi.it; 3 Centre Scientifique de Monaco, 8 Quai Antoine 1°, 98000 Monaco, Monaco; zoccola@centrescientifique.mc (D.Z.); stambutte@centrescientifique.mc (S.T.); 4 Dipartimento Neurofarba, Sezione di Scienze Farmaceutiche e Nutraceutiche, Università degli Studi di Firenze, Via U. Schiff 6, 50019 Sesto Fiorentino (Florence), Italy

**Keywords:** carbonic anhydrase, activators, biomineralization, coral, calcification, amine, amino acid

## Abstract

CruCA4, a coral α-carbonic anhydrase (CA, EC 4.2.1.1) involved in the biomineralization process of the Mediterranean red coral, *Corallium rubrum*, was investigated for its activation with a panel of amino acids and amines. Most compounds showed considerable activating properties, with a rather well defined structure–activity relationship. The most effective CruCA4 activators were d-His, 4-H_2_N-l-Phe, Histamine, Dopamine, Serotonin, 1-(2-Aminoethyl)-piperazine, and l-Adrenaline, with activation constants in the range of 8–98 nM. Other amines and amino acids, such as d-DOPA, l-Tyr, 2-Pyridyl-methylamine, 2-(2-Aminoethyl) pyridine and 4-(2-Aminoethyl)-morpholine, were submicromolar CruCA4 activators, with K_A_ ranging between 0.15 and 0.93 µM. Since it has been shown that CA activators may facilitate the initial phases of in-bone mineralization, our study may be relevant for finding modulators of enzyme activity, which can enhance the formation of the red coral skeleton.

## 1. Introduction

Carbonic anhydrases (CAs, EC 4.2.1.1) are metalloenzymes, which catalyze the reversible hydration reaction of carbon dioxide to bicarbonate (HCO_3_^−^) and protons (H^+^) [1,2,3]. This simple but physiologically crucial reaction is essential for maintaining the metabolic balance of the inorganic carbon in all living organisms [4,5,6,7,8,9]. CAs are characterized by a catalytic constant (k_cat)_ value ranging from 10^4^ to 10^6^ s^−1^ for supporting the fastest metabolic activities of the cell, such as the transport and secretory processes of the inorganic carbon [1,3,7,8]. The natural spontaneous CO_2_ hydration/dehydration reaction has a first-order rate constant of 0.15 s^–1^ [8,10,11,12,13,14]. In 1940, an emerging and interesting feature of CAs was discovered: the velocity of this already fast enzyme could be improved by the use of small molecules [15,16]. These molecules were named CA activators (CAAs) and were identified to belong to various chemical classes, such as biogenic amines (histamine, serotonin, and catecholamines), amino acids, oligopeptides, or small proteins [17,18,19,20]. In the initial phases of this research, the CAAs’ discovery created a controversy among the scientists because some of them supported the thesis that CAAs did not exist, whereas others even reinforced the idea that CA activation was an artifact [21,22,23]. These controversies contributed to a slow progress in the field of CAAs. Thus, the scientific research on CAs steadfastly progressed with new findings regarding their catalytic and inhibition mechanisms, but it was necessary to wait until the early 1990’s for a completely different perception of the CAAs [15,16,24]. Only in 1997, with the report of the first crystallographic structure of human (h) isoform hCA II complexed with different activators was the effectiveness of CAAs definitively proven. In addition, the possibility that they could strongly enhance the enzyme activity was recognized, and thus the idea to design new pharmacological or environmental applications for this class of modulators of activity was finally considered [25,26]. Today, through the extensive use of X-ray crystal structure, spectroscopic and kinetic data, it is accepted that CAAs are molecules which are able to bind within the active site in various regions of the cavity, but far away enough from the metal ion with which they do not directly interact. Furthermore, the CAAs do not influence the binding of CO_2_ to the enzyme, but make easier the transfer of protons from the active site to the environment, increasing the catalytic constant of the enzyme [17,19,21,25,26,27,28,29,30,31]. Intriguingly, in the last decade, the CAAs, which were considered for long time as molecules of doubtful relevance, acquired an enormous importance for some pharmacological applications. For example, several human diseases, such as osteopetrosis, cerebral calcifications, retinal problems, hyperammonemia, and hyperchlorhidrosis, are characterized by a deficiency in the activity of several human CA isoforms [21,29,30,31,32]. Thus, it is immediately apparent that the aforementioned human disorders could be treated by using CAAs. Furthermore, it has been demonstrated that CAAs can improve synaptic efficacy, spatial learning, and memory [18,33,34]. Recently, as described in the literature, a new and interesting aspect of CAAs has emerged. These molecules are capable of enhancing the formation of the inorganic salts, such as calcium carbonate and calcium phosphate, which are involved in the biomineralization process [29,30,32]. Biomineralization activity is typical of all the organisms characterized by a shell or bones/skeletons [35]. The organism produces minerals such as silicates in algae and diatoms, carbonates in invertebrates, calcium phosphates and carbonates in vertebrates [35]. In this context, we have here investigated in vitro the effect of CAAs (amino acids and amines) on the activity of the recombinant CruCA4 from Mediterranean red coral, *Corallium rubrum*. CruCA4 belongs to the α-CA class and is an enzyme with a significant hydratase activity involved in coral skeleton formation [36,37,38]. At the site of calcification, CruCA4 generates bicarbonate which is then converted into carbonate [37,38]. The carbonate reacts with calcium in order to precipitate as calcium carbonate (CaCO_3_), which is responsible for coral skeleton formation [36,37,38,39]. Our in vitro results demonstrate that CruCA4 is effectively activated by several amino acids and amines considered in the present study. It is interesting to note that the cnidarian coral skeleton represents a good example of biomedical material because its structure and architecture are similar to that of bones [35]. Since red corals grow very slowly, about few centimeters per year, one of the possible findings of this paper raises the possibility of using CAAs for enhancing the in vivo growth rates of coral skeletons.

## 2. Results and Discussion

### 2.1. CruCA4 Identification, Heterologous Expression, Purification, and Kinetic Analysis

The inspection of the *C. rubrum* genome and transcriptome highlighted the presence of six α-CA isoforms, named with the acronym CruCA and Arabic numerals 1 to 6 (Table 1). Their nucleotide sequences consist of an open reading frame encoding for a polypeptide chain ranging from 263 to 356 amino acid residues (Table 1). The theoretical molecular weight of all CruCA isoforms ranged between 29.0 and 41.0 kDa, as determined using the program Compute MW (https://web.expasy.org/compute_pi/) [40] (Table 1). 

Intriguingly, as demonstrated by our groups, even if CruCA4 was expressed at very low levels with respect to the other isoforms, it was the only isoenzyme expressed in the tissue-calcifying fraction [37,38]. Thus, CruCA4 attracted our attention as the main coral α-CA involved in the biomineralization process. The recombinant CruCA4 was prepared by designing a synthetic gene lacking the signal peptide responsible for CruCA4 secretion. The expression vector was composed of a chimeric gene resulting from the fusion of the CruCA4 gene having a nucleotide tail encoding for six residues of histidine at the 5′ extremity. The recombinant enzyme was heterologously expressed in *Eschericia coli*. Our results demonstrated that the enzyme was recovered in the soluble fraction of the *E. coli* cell extract and purified to an apparent homogeneity by a nickel affinity gel, as demonstrated by SDS-PAGE (data non shown). The recovered amount of the enzyme was about 1 mg, starting from 1 L of cellular bacterial culture. 

The CruCA4 kinetic constants were determined using the stopped-flow technique. The enzyme had high catalytic activity for the physiological reaction of CO_2_ hydration to bicarbonate and protons, with a k_cat_ of 2.4 × 10^5^ s^–1^ and a catalytic efficiency (k_cat_/K_M_) of 5.2 × 10^7^ M^–1^ s^–1^. These results are very interesting because the high catalytic efficiency of the isoenzyme compensates for the low level of expression of CruCA4 in the tissue-calcifying fraction, underlining the importance of CruCA4 in the biomineralization process of the red coral.

### 2.2. CruCA4 Activation Profile

**Figure 1:**
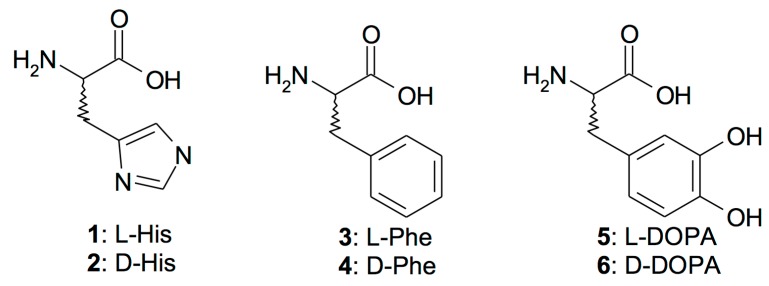

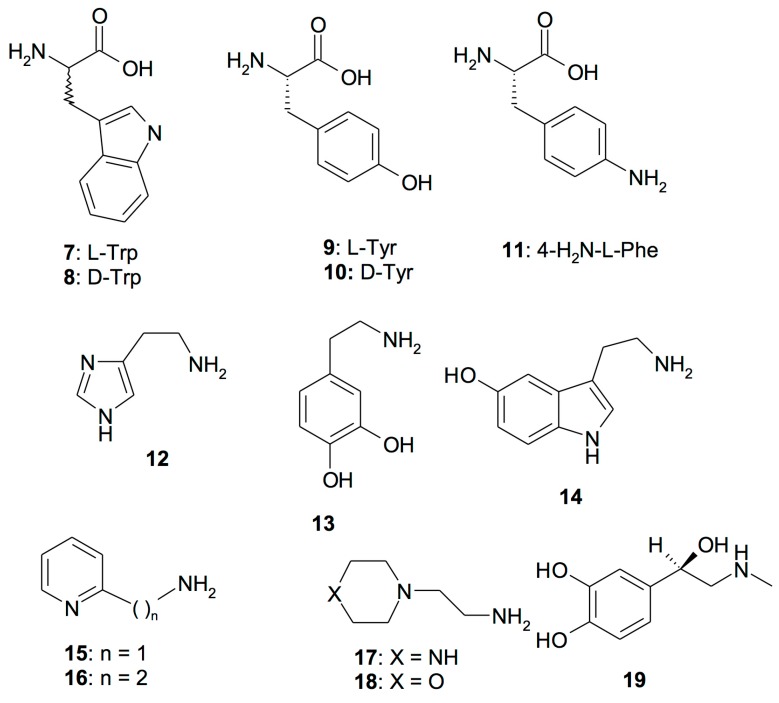
Amino acids **1**–**11** and amines **12**–**19** investigated as CruCA4 activators.

As deduced from the primary structure, the CruCA4 amino acid sequence contains most of the typical features of an α-CA, such as the three histidine residues, involved in the metal-coordination, and the two gatekeeper residues, involved in the binding of inhibitors. CruCA4 lacks the proton shuttle residue His64 (following the hCAI numbering system), the residue involved in the restoration of the catalytic active form of the enzyme through the transfer of a proton from the water coordinated Zn(II) ion to the environment. CruCA4 has a residue of Lys instead of His in position 64. This substitution, as demonstrated for other CAs, leads to a decrease in the k_cat_ value [41,42]. Thus, the treatment of the enzyme with CAAs represents a very good strategy for stimulating the biomineralization process of the organism through the increase of the enzymatic activity of CruCA4. We want to stress the fact that this enzyme is involved in the formation of the coral skeleton. Thus, activators **1**–**19** have been investigated as CAAs to be used in conjunction with CruCA4 (Figure 1). Figure 1 includes both natural and non-natural amino acids and amines.

As seen from the data of Table 2, l-Tyr, one of the effective CruCA4 activators examined here, did not change the K_M_ of the enzyme but had a notable effect on k_cat_, which is consistent with observations of activators of other CAs [43,44,45,46]. In fact, 10 µM l-Tyr produced a four-fold enhancement of k_cat_ compared to the value of this parameter in the absence of the activator. The same effects were observed for human isoforms hCA I and II, which are significantly activated by l-Tyr (Table 2).

Amino acids and amines **1**–**19** (Figure 1) can potently activate CruCA4, with many activators having been identified that have activation constants in the nanomolar range (Table 3). For comparison, K_A_ values for hCA I and II are also reported in Table 3. The following structure–activity relationship (SAR) observations were obtained from the data of Table 3.

**1.** The most effective CruCA4 activators were d-His (**2**), 4-H_2_N-l-Phe (**11**), Histamine (**12**), Dopamine (**13**), Serotonin (**14**), 1-(2-Aminoethyl)-piperazine (**17**), and l-Adrenaline (**19**), which have activation constants in the range of 8–98 nM. It can be observed that amine type compounds are more effective CruCA4 activators (K_A_ values ranging from 5 to 9 nM) compared to the natural and non-natural amino acids (K_A_ values ranging from 74 to 98 nM).

**2.** Most of the amines and amino acids **1**–**19** were effective submicromolar CruCA4 activators, with K_A_ values ranging between 0.15 and 0.93 µM. They include d-DOPA (**6**), l-Tyr (**9**), 2-Pyridyl-methylamine (**15**), 2-(2-Aminoethyl)pyridine (**16**) and 4-(2-Aminoethyl)-morpholine (**18**) (Table 2). 

**3.** Except for l-Tyr (**9**) and d-Tyr (**10**), in which the l-enantiomer was a more effective activator than the d-enantiomer, the d-enantiomers were generally more effective activators of CruCA4 compared to their corresponding l-enantiomers. As demonstrated by X-ray crystallographic studies on human isoforms hCA I and II [44,45,46], l- and d-enantiomers can bind differently within the active site binding pocket owing to the different stereochemistry induced by the asymmetric carbon atom. This can result in different activating properties, as seems to also be the case for the coral enzyme.

**4.** The least effective CruCA4 activators were l-His (**1**), l-/d-Phe (**3,4**), L-DOPA (**5**), l-/d-Trp (**7,8**), and d-Tyr (**10**), which have relatively limited potency as activators (K_A_ values of 1.01 to 36.9 µM) (Table 2).

**5.** An important difference in the activation profile of CruCA4 compared to the human isoforms hCA I and II was observed for amines and amino acids **1**–**19** (Table 2). For example, Histamine (**12**), Dopamine (**13**), Serotonin (**14**), 1-(2-Aminoethyl)-piperazine (**17**) and l-Adrenaline (**19**) seemed to be highly selective for CruCA4, being low nanomolar CruCA4 activators, but also produced activity in the high micromolar range for hCA I and II. Probably, these activators are more specific to the coral enzyme because CruCA4 clusterizes in a distinct group, which is different from the two human isoforms hCA I and hCA II, as demonstrated by the phylogenetic analysis carried out on cnidarian and human α-CAs [38].

## 3. Materials and Methods

### 3.1. Chemistry

Amino acids and amines **1**–**19** were the commercially available, highest purity reagents from Sigma-Aldrich, Milan, Italy. All other chemicals used were from Sigma-Aldrich as well.

### 3.2. Gene Synthesis, Cloning, Expression, Purification

The GeneArt Company (Thermo Fisher Scientific, Waltham, MA, USA), specialized in gene synthesis, designed the synthetic CruCA4 gene (Accession number: KU557746.1) without the peptide signal and with the four base-pair sequences (CACC) at the 5′ end which are necessary for directional cloning in the pMK-T vector (subcloning vector, Thermo Fisher Scientific). The CruCA4 was subsequently cloned into the expression vector pET100/D-TOPO (Invitrogen, Carlsbad, CA, USA), creating the plasmid pET100D-Topo/CruCA4 and containing a nucleotide sequence encoding for a peptide containing six histidines before the insertion point, for facilitating the purification of the target protein. In order to confirm the gene integrity and that no errors occurred at the ligation sites, the vector containing the fragment was subject to bidirectional automated sequencing. 

Escherichia coli ArcticExpress (DE3)RIL competent cells were transformed with pET100D-Topo/CruCA4, grown at 20 °C and induced with 1 mM IPTG. ZnSO_4_ was added after 30 min and after 3 h of additional growth, cells were harvested and disrupted by sonication at 4 °C in 20 mM buffer phosphate, pH 8.0. Following sonication, the sample was centrifuged at 1200× *g* at 4 °C for 30 min. The supernatant was dialyzed at 4 °C against 0.02 M phosphate buffer (pH 8.0) containing 0.01 M imidazole, and loaded onto a His-select HF Nickel affinity column (GE Healthcare, dimension: 1.0 × 10 cm). The column was equilibrated with 0.02 M phosphate buffer (pH 8.0) containing 0.01 M imidazole and 0.5 M KCl at a flow rate of 1.0 mL/min. The recombinant CgiNAP2X1 was eluted from the column with 0.02 M phosphate buffer (pH 8.0) containing 0.5 M KCl and 0.3 M imidazole at a flow rate of 1.0 mL/min. Active fractions (0.5 mL) were collected and combined to a total volume of 2.5 mL. Subsequently, they were dialyzed, concentrated and analyzed by SDS-PAGE. At this stage of purification, the enzyme was at least 95% pure and the amount obtained was 1.0 mg.

### 3.3. SDS-PAGE 

Sodium dodecyl sulfate SDS-polyacrylamide gel electrophoresis (SDS-PAGE) was performed as described by Laemmli using 12% gels [47]. 

### 3.4. CA Enzyme Activation Assay

An Sx.18Mv-R Applied Photophysics (Oxford, UK) stopped-flow instrument was used to assay the catalytic activity of various CA isozymes for CO_2_ hydration reaction [48]. Phenol red (at a concentration of 0.2 mM) was used as indicator, working at the absorbance maximum of 557 nm, with 10 mM Hepes (pH 7.5) or TRIS (pH 8.3) as buffers, and 0.1 M Na_2_SO_4_ (for maintaining constant ionic strength), following the CA-catalyzed CO_2_ hydration reaction for a period of 10 s at 25 °C. Activity of the α-CA was measured at pH 7.5, as it has been reported that this is the optimal pH value for enzymes of this class [49,50,51]. The CO_2_ concentrations ranged from 1.7 to 17 mM for the determination of the kinetic parameters and activation constants. For each activator, at least six traces of the initial 5–10% of the reaction were used for determining the initial velocity. The uncatalyzed rates were determined in the same manner and subtracted from the total observed rates. Stock solutions of activators (10 mM) were prepared in distilled-deionized water and dilutions of up to 1 nM were done thereafter with the assay buffer. Activator and enzyme solutions were pre-incubated together for 15 min (standard assay at room temperature) prior to assay, in order to allow for the formation of the E–A complex. The activation constant (K_A_), defined similarly with the inhibition constant K_I_, can be obtained by considering the classical Michaelis–Menten equation (Equation (1)), which has been fitted by non-linear least squares by using PRISM 3:

v = v_max_/{1 + K_M_/[S](1 + [A]_f_/K_A_)} (1)

where [A]_f_ is the free concentration of activator.

Working at substrate concentrations considerably lower than K_M_ ([S] << K_M_), and considering that [A]_f_ can be represented in the form of the total concentration of the enzyme ([E]_t_) and activator ([A]_t_), the obtained competitive steady-state equation for determining the activation constant is given by Equation (2) [24,25]:

v = v_0_K_A_/{K_A_ + ([A]_t_ − 0.5{([A]_t_ + [E]_t_ + K_A_) − ([A]_t_ + [E]_t_ + K_A_)^2^ − 4[A]_t_[E]_t_)^1/2^}} (2)

where v_0_ represents the initial velocity of the enzyme-catalyzed reaction in the absence of activator.

## 4. Conclusions

We report here an activation study of the coral α-CA, CruCA4, involved in mineralization, which was cloned and characterized by us from the Mediterranean red coral, *Corallium rubrum*. A panel of amino acids and amines showed considerable activating properties, with a rather well defined structure–activity relationship. Thus, effective CruCA4 activators were identified, such as D-His, Histamine, Dopamine, etc. with activation constants in the range of 8–98 nM. Other amines and amino acids, such as D-DOPA, l-Tyr, 2-Pyridyl-methylamine, 2-(2-Aminoethyl)pyridine and 4-(2-Aminoethyl)-morpholine were submicromolar CruCA4 activators, with K_A_ values ranging between 0.15 and 0.93 µM. Since Muller’s group [52,53] has shown that CAAs may facilitate processes in other biological systems, for example the first phases of bone mineralization, our study may be relevant for finding modulators of enzyme activity, which could enhance the formation of the red coral skeleton.

## Figures and Tables

**Table 1:** Features and physiological localization of the α-CAs identified in the genome of the Mediterranean red coral, *Corallium rubrum*.

Acronym	GenBank Accession Number	Amino Acid Number	M.W. (kDa)	Calcifying Fraction ^1^	Non-Calcifying Fraction ^1^
**CruCA1**	KU557743.1	356	40.89	−	+
**CruCA2**	KU557744.1	322	36.90	−	+
**CruCA3**	KU557745.1	262	29.04	−	+
**CruCA4**	KU557746.1	284	32.86	+	−
**CruCA5**	KU557747.1	335	36.06	−	+
**CruCA5**	KU557748.1	281	32.06	−	+

^1^ Data from Le Goff and coworkers [38]. M.W. is molecular weight.

**Table 2:** Activation of human carbonic anhydrase (hCA) isozymes I, II, and CruCA4 with l-Tyr, at 25 °C, for the CO_2_ hydration reaction.

Isoenzyme	k_cat_ * (s^−1^)	K_M_ * (mM)	(k_cat_)_l-Tyr_ ** (s^−1^)	K_A_ *** l-Tyr (μM)
hCA I ^a^	2.0 × 10^5^	4.0	13.9 × 10^5^	0.020
hCA II ^a^	1.4 × 10^6^	9.3	12.8 × 10^6^	0.011
CruCA4 ^b^	2.4 × 10^5^	4.6	18.7 × 10^5^	0.73

* Observed catalytic rate without activator. K_M_ values in the presence and the absence of activators were the same for the various CAs (data not shown); ** Observed catalytic rate in the presence of 10 μM of the activator; *** The activation constant (KA) for each enzyme was obtained by fitting the observed catalytic enhancements as a function of the activator concentration. Data represents mean from at least three determinations by a stopped-flow, CO_2_ hydrase method. Standard errors were in the range of 5–10% of the reported values (data not shown). ^a^ Human recombinant isozymes, from Ref. [46]; ^b^ Coral recombinant enzyme, from this work.

**Table 3:** Activation constants of hCA I, hCA II and the coral CruCA4 with amino acids and amines **1**–**19**. Data for hCA I and II are from Di Cesare and colleagues [43].

No.	Compound	K_A_ (μM) *		
		hCA I ^a^	hCA II ^a^	CruCA4 ^b^
**1**	l-His	0.03	10.9	36.9
**2**	d-His	0.09	43	0.098
**3**	l-Phe	0.07	0.013	15.4
**4**	d-Phe	86	0.035	1.01
**5**	l-DOPA	3.1	11.4	13.7
**6**	d-DOPA	4.9	7.8	0.93
**7**	l-Trp	44	27	9.48
**8**	d-Trp	41	12	8.35
**9**	l-Tyr	0.02	0.011	0.73
**10**	d-Tyr	0.04	0.013	18.9
**11**	4-H_2_N-l-Phe	0.24	0.15	0.074
**12**	Histamine	2.1	125	0.007
**13**	Dopamine	13.5	9.2	0.005
**14**	Serotonin	45	50	0.006
**15**	2-Pyridyl-methylamine	26	34	0.41
**16**	2-(2-Aminoethyl)pyridine	13	15	0.26
**17**	1-(2-Aminoethyl)-piperazine	7.4	2.3	0.004
**18**	4-(2-Aminoethyl)-morpholine	0.14	0.19	0.15
**19**	l-Adrenaline	0.09	96	0.009

* Data represents mean from three determinations by a stopped-flow, CO_2_ hydrase method. Standard errors were in the range of 5–10% of the reported values (data not shown); ^a^ Human recombinant isozymes, stopped flow CO_2_ hydrase assay method [5]; ^b^ From this work.
